# Nitrogen- and oxygen-rich organic material indicative of polymerization in pre-aqueous cryochemistry on Bennu’s parent body

**DOI:** 10.1038/s41550-025-02694-5

**Published:** 2025-12-02

**Authors:** Scott A. Sandford, Zack Gainsforth, Michel Nuevo, Matthew A. Marcus, Hans A. Bechtel, Ryan C. Ogliore, Clive Jones, Gerardo Dominguez, Daniel P. Glavin, Jason P. Dworkin, Timothy J. McCoy, Sara S. Russell, Thomas J. Zega, Harold C. Connolly, Dante S. Lauretta

**Affiliations:** 1 Space Science and Astrobiology Division, NASA Ames Research Center (ARC), Moffett Field, CA USA; 2 Space Sciences Laboratory, University of California, Berkeley, CA USA; 3 Advanced Light Source Division, Lawrence Berkeley National Laboratory, Berkeley, CA USA; 4 Department of Physics, Washington University in St Louis, St Louis, MO USA; 5 Department of Earth and Planetary Sciences, Washington University in St Louis, St Louis, MO USA; 6 Department of Physics, California State University San Marcos, San Marcos, CA USA; 7 Solar System Exploration Division, NASA Goddard Space Flight Center (GSFC), Greenbelt, MD USA; 8 Department of Mineral Sciences, National Museum of Natural History, Smithsonian Institution, Washington, DC USA; 9 Planetary Materials Group, Natural History Museum, London, UK; 10 Lunar and Planetary Laboratory, University of Arizona, Tucson, AZ USA; 11 Department of Geology, School of Earth and Environment, Rowan University, Glassboro, NJ USA; 12 Department of Earth and Planetary Science, American Museum of Natural History, New York, NY USA

**Keywords:** Asteroids, comets and Kuiper belt, Astrobiology, Meteoritics, Geochemistry

## Abstract

Nitrogen-containing organic compounds play key biological roles, and their identification in primitive astromaterials such as meteorites can shed light on the origin of life. However, meteorites are typically contaminated by uncontrolled exposure to Earth. Here we show that pristine samples returned from asteroid Bennu contain polymeric organics exceptionally rich in nitrogen and oxygen. These polymers contain a variety of functional groups including amines, amides, N-heterocycles, and aliphatic and aromatic hydrocarbons, among others. They are seen in a carbonaceous vein with mineral inclusions and in multilayered organic sheets. Their morphology and composition indicate formation from pre-aqueous N-rich precursors and later modification during aqueous alteration. These findings demonstrate that asteroids like Bennu contain complex nitrogen-rich organic phases formed by pre-aqueous and aqueous processes, and they expand the known inventory of potential prebiotic extraterrestrial compounds.

## Abstract (web summary)

Samples returned from asteroid Bennu by NASA’s OSIRIS-REx mission contain N-rich organics with prebiotic implications. This material probably formed in the earliest stages of the asteroid’s history possibly even before its water ice melted.

## Main

NASA’s Origins, Spectral Interpretation, Resource Identification, and Security–Regolith Explorer (OSIRIS-REx) spacecraft returned 121.6 g of pristine regolith from asteroid (101955) Bennu to Earth^1,2^. Bennu is an ~500-m-diameter near-Earth rubble pile^3^, probably originating from a parent asteroid large enough to support a hydrothermal system before its catastrophic destruction^4,5^. OSIRIS-REx observations corroborated predictions of a hydrated, carbon-rich composition^6,7^. Infrared (IR) observations showed evidence for abundant organic compounds, including aliphatic carbon exhibiting a 3.4-µm C–H stretching mode^8^. An N–H stretching mode at 3.1 µm in some Bennu spectra indicate the presence of phases such as ammonium salts or N-rich organic matter^9^.

Analyses of the returned samples confirmed that they have been extensively altered by interactions with aqueous fluids and are like the Ivuna-type (CI) carbonaceous chondrites^2,10,11^, with a bulk C/N ratio of ~20 (ref. ^2^). Ammonia (NH_3_) and N-rich organic compounds, including amines, amino acids and N-heterocycles, were found in the Bennu soluble organic fraction^12^.

Here we describe a polymeric organic phase discovered in Bennu particles that is exceptionally rich in nitrogen and oxygen. We use Fourier-transform infrared (FTIR) spectroscopy, scanning transmission X-ray microscopy (STXM), scanning electron microscopy (SEM), transmission electron microscopy (TEM) and secondary-ion mass spectroscopy (SIMS) to characterize this notable carbonaceous material.

## Results

### Infrared spectroscopy

**Extended Data Fig. 1:**
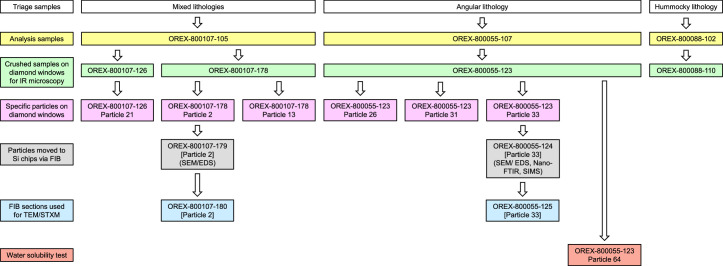
Sample preparation flowchart showing the sample IDs and techniques used to study them. The individual Particle 2 (from mixed sample OREX-800107-178) and Particle 33 (from angular lithology OREX-800055-123), first studied with IR microscopy, were isolated, transferred to different substrates, and analyzed with additional techniques (SEM, EDS, nano-FTIR, SIMS, TEM, and STXM).

We measured the mid-IR spectra (4,000–675 cm^−1^) of a powdered Bennu material sampling the angular lithology (OREX-800055-107), a powder sampling the hummocky lithology (OREX-800088-102) and a powder sampling an unsorted mixture of particles <0.5 cm, ostensibly comprising angular, hummocky and potentially other lithologies (OREX-800107-105; henceforth termed the ‘mixed sample’). The lithologies are described elsewhere^2,10^. Submilligram portions of each sample were prepared for FTIR analysis on diamond window substrates (Table 1 and Extended Data Fig. 1).

**Table 1:** Summary of particles examined in this study

Parent sample ID and lithology	Subsample ID	Particle ID	Organic composition	Petrography	Techniques
OREX-800107-105, mixed	OREX-800107-178	Particle 2^a^	N- and O-rich organics, C:N:O ratio (3:1:1)	Organic vein sandwiched between phyllosilicates; associated with carbonate inclusions	FTIR, SEM-EDS, STEM, STXM
OREX-800107-105, mixed	OREX-800107-178	Particle 13^b^	None	Carbonate-dominated grain	FTIR
OREX-800107-105, mixed	OREX-800107-126	Particle 21^b^	Minor abundance of N-poor organics, very weak aliphatic bands	Common spectral type, dominated by phyllosilicates and carbonates	FTIR
OREX-800055-107, angular	OREX-800055-123	Particle 26^a^	N-rich organics, highest CH_2_/CH_3_ ratio seen for N-rich organics, possible esters or lactones	Organic phase seen in association with phyllosilicates and carbonates	FTIR
OREX-800055-107, angular	OREX-800055-123	Particle 31^b^	Minor abundance of N-poor organics, weak aliphatic bands	Common spectral type, dominated by phyllosilicates with various carbonates; occurs within the same ~1-cm rock as the N-rich organics found in Particle 33	FTIR
OREX-800055-107, angular	OREX-800055-123	Particle 33^a,b^	N- and O-rich organics, C:N:O ratio (5:1:1)	Multilayered organic sheet free of associated phyllosilicates; contains carbonate inclusions; transparent in optical	FTIR, nano-FTIR, SEM-EDS, STEM, STXM
OREX-800055-107, angular	OREX-800055-123	Particle 64	N- and O-rich organics	Organic phase seen in association with phyllosilicates	Water solubility test
OREX-800088-102, hummocky	OREX-800088-110	N/A	Minor abundance of N-poor organics, weak aliphatic bands	Common spectral type, dominated by phyllosilicates with various carbonates	FTIR

^a^FTIR spectrum shown in Fig. 1. ^b^FTIR spectrum shown in Extended Data Fig. 2. N/A, not applicable.

**Fig. 1:**
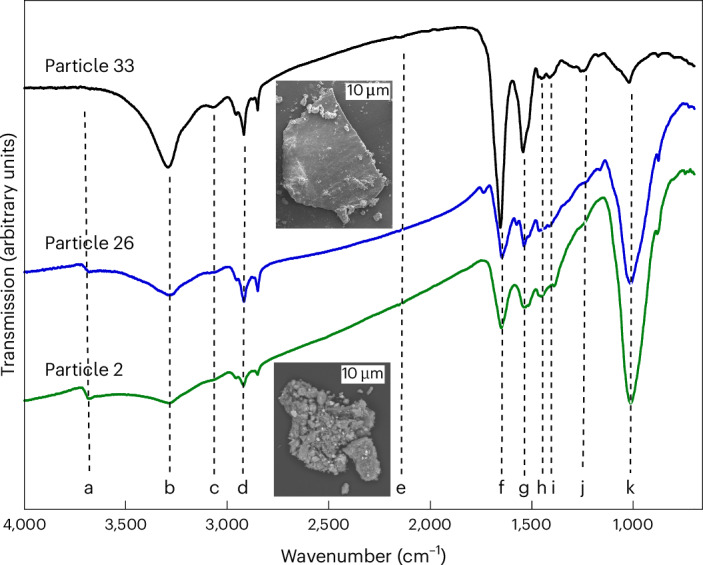
The mid-IR spectra of particles that contain nitrogen-rich organics. The bottom two spectra are from particles that contained associated phyllosilicates and carbonates. The top spectrum contains very little phyllosilicate. The spectral bands are as follows: structural –OH at 3,685 cm^−1^ (a), N–H stretch at 3,285 cm^−1^ (b), a weak aromatic C–H stretch near 3,065 cm^−1^ (c), aliphatic C–H stretch is a set of bands in the 3,000–2,820 cm^−1^ range (d), a weak C≡N peak is visible at 2,145 cm^−1^ (e), amide C=O stretch at 1,650 cm^−1^ (f), N–H bend at 1,540 cm^−1^ (g), CH_2_ bend and C–O stretch in carbonates at 1,465 cm^−1^ (h), C–N stretch at 1,410 cm^−1^ (i), C–O stretch in aromatic ethers and CH_3_ bending in the 1,255–1,240 cm^−1^ range (j), Si–O stretch in phyllosilicates at 1,015 cm^−1^ (k). Peak positions and assignments are summarized in Table 2. Insets: SEM images of Particles 2 and 33. Particle 26 was not analysed by SEM.

**Extended Data Fig. 2:**
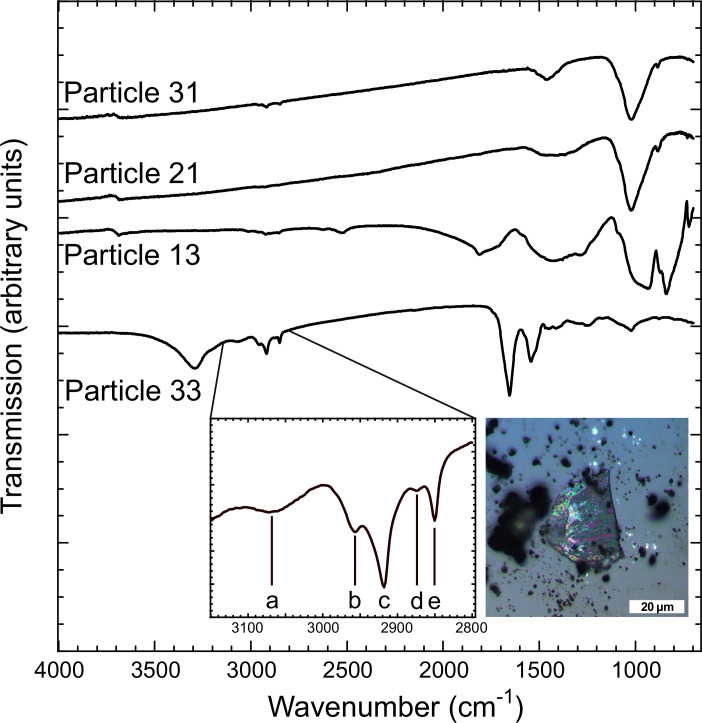
Comparison of the infrared spectra of the N-rich carbonaceous material with other spectra more commonly found in samples OREX-800107-126 and OREX-800107-178 (mixed lithologies) and OREX-800055-123 (angular lithology). The top two spectra, for OREX-800055-123 Particle 31 and OREX-800107-126 Particle 21, are representative of the majority of the particles examined in all the studied samples. They are dominated by the strong Si–O stretch feature of phyllosilicates near 1015 cm^−1^, and show a weaker feature near 3285 cm^−1^ due to –OH bridging groups within phyllosilicates. The weak features between 3000 and 2820 cm^−1^ are due to aliphatic –CH_2_– and –CH_3_ groups, where –CH_2_– groups dominate. The spectra of most particles dominated by phyllosilicates show a weak feature near 880 cm^−1^ and broad absorption, usually centered near 1465 cm^−1^, that indicates the presence of carbonates. Occasional grains such as OREX-800107-178 Particle 13 seem to consist predominantly or solely of carbonate minerals, and their spectra show the characteristic vibrational fundamental band near 1425 cm^−1^ (CO_3_ asymmetric stretch, ν_3_) and a series of overtone/combination modes at 3015, 2965–2850, 2625, 2520, and 1810 cm^−1^. The spectra of the new, N-rich organic phase (bottom spectrum, OREX-800055-123 Particle 33) contrasts starkly with the more typical spectra seen from other particles in our Bennu samples (Fig. 1 and Table 2). The spectral insert shows a magnification of the spectrum of Particle 33 in the 3150–2800 cm^−1^ range that covers the C–H stretch mode region. Bands are seen due to the aromatic C–H stretch (a), as well as aliphatic –CH_3_ asymmetric (b), –CH_2_– asymmetric (c), –CH_3_ symmetric (d), and –CH_2_– symmetric (e) stretches. Also inserted is an optical image of Particle 33 on a diamond substrate showing interference fringes. The original particle was a thin, clear organic sheet.

**Extended Data Fig. 3:**
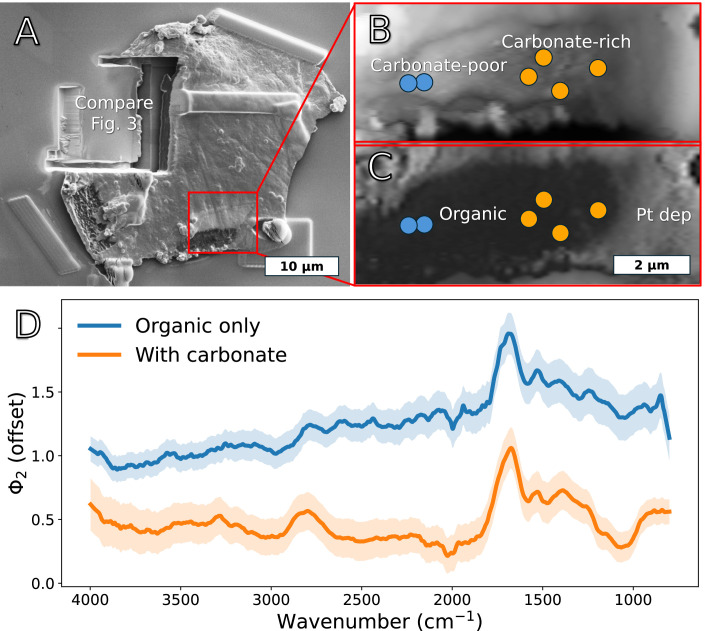
Nano-FTIR analysis of Particle 33. **A**, SEM image of Particle 33 showing where the FIB was used to mill through the protective Pt (box in red). The location FIBed for 3D reconstruction (Fig. 3) and STXM/TEM can also be seen in the upper left quadrant. **B**, Nano-FTIR map showing the sample height as a function of position. The ion beam milled deeper on the right side of the patch and exposed subsurface carbonates. Spectra from four locations (orange circles) were averaged to produce the orange spectrum shown in **D**. Spectra from two locations (blue circles) were averaged to produce the blue spectrum in **D**. **C**, S_2_, the integrated infrared scattering intensity. The regions coated in Pt are brighter than the organic material. **D**, Spectra showing carbonate-rich (orange) and carbonate-poor (blue) organic spectra. The bold lines are the mean spectra with the 1σ standard deviation of the averaged spectra is plotted in a lighter shade. The N–H bend and amide C=O stretch peaks are visible at 1540 cm^−1^ and 1650 cm^−1^, respectively, with a blue-shift of several cm^−1^ from the near-field interaction. A carbonate peak at 1465 cm^−1^ and overtones near 2800 cm^−1^ are visible in the orange spectrum. The N–H stretch at 3285 cm^−1^ is visible in both spectra. The Si–O feature at 1015 cm^−1^ is absent in both spectra.

The IR spectra of most powder particles are dominated by phyllosilicate-carbonate mixtures. Many particles contain organics, evidenced by C–H stretching bands in the 3,000–2,820 cm^−1^ range. The dominant C–H stretching modes are from aliphatic functional groups, although weak aromatic C–H stretching features around 3,065 cm^−1^ are occasionally seen. The aliphatic features show the presence of CH_2_ and CH_3_ groups, with CH_2_ groups dominating (Fig. 1 and Extended Data Fig. 2). Nano-FTIR results confirm that these functional groups are associated with the organic phase and not the phyllosilicate (Extended Data Fig. 3).

**Fig. 2:**
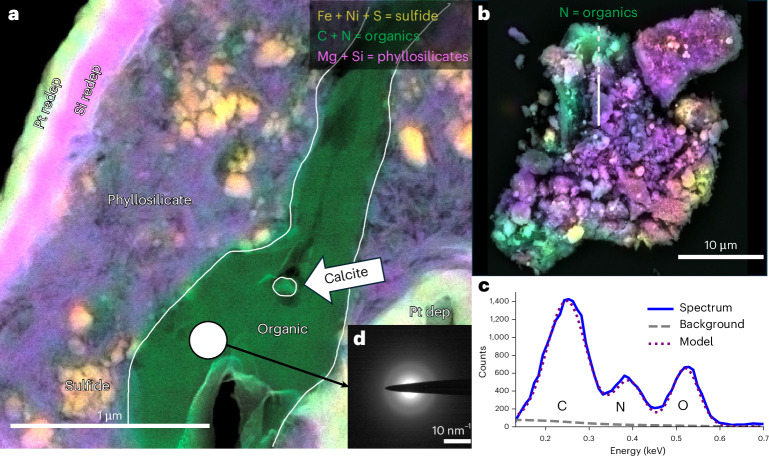
Electron microscopy of Particle 2. **a**, STEM high-angle annular dark field (HAADF) + EDS image of Particle 2. A nitrogenous vein (green) is sandwiched between phyllosilicate regions (purple). A circular region shows the location of a selected area electron diffraction acquisition shown in **b**. The Pt and Si deposition and redeposition layers were created during the FIB processing. **b**, Selected area electron diffraction shows that the nitrogenous vein is amorphous. **c**, SEM backscatter plus EDS view of the larger particle before the FIB section was extracted. The substrate is diamond; only N was used for the green channel. The line shows the location of the FIB liftout; the dashed portion of the line is outside the field of view in **a**. **d**, EDS spectrum of the CNO peaks of the organic phase along with the fit used for quantification. dep, deposition; redep, redeposition.

**Fig. 3:**
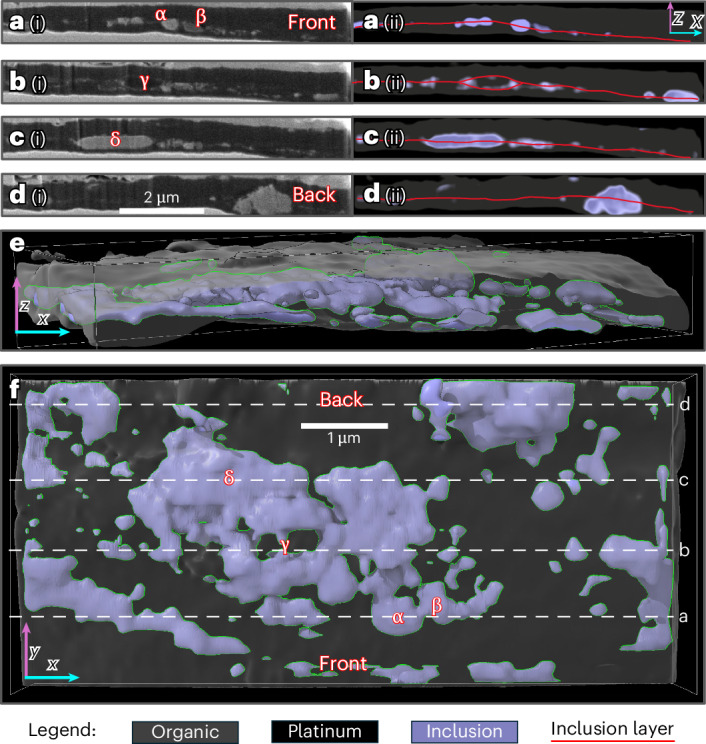
A 3D reconstruction of a FIB-extracted portion of Particle 33. **a**(i)–**d**(i) Secondary electron images of the exposed surface during a slice-and-view operation in the FIB, viewing the sample edge on. The reconstruction was made from slices cut approximately 100 nm deeper than the last. These images are in the *xz* plane of the 3D reconstruction. Each panel shows the sandwich structure of the particle with protective platinum (brightest) on the top and bottom. Organic is dark, and inclusions have a moderate brightness and appear as an inclusion layer. α and β denote two side-by-side grains with differing secondary electron contrasts, and therefore differing compositions within the inclusion layer. γ denotes a pocket of organics within the inclusion layer. δ denotes a large euhedral inclusion, about 1.75 μm long. **a**(ii)–**d**(ii), The same slices as **a**(i)–**d**(i) viewed in the 3D reconstruction. Superimposed lines trace the approximate position of the inclusion layer, which is frequently offset from the midline. **e**, Orthographic projection of the reconstruction near the *xz* plane. **f**, Top-down view of the *xy* plane of the reconstruction with α–δ labelled as before. Random clumping of the inclusions is apparent. The dashed lines labelled **a**–**d** in **f** show the locations of the slices in **a**(ii)–**d**(ii). Thin green lines in **e** and **f** delineate the outline of the inclusion phases. See also Supplementary Videos 1 and 2 for raw data.

**Extended Data Fig. 4:**
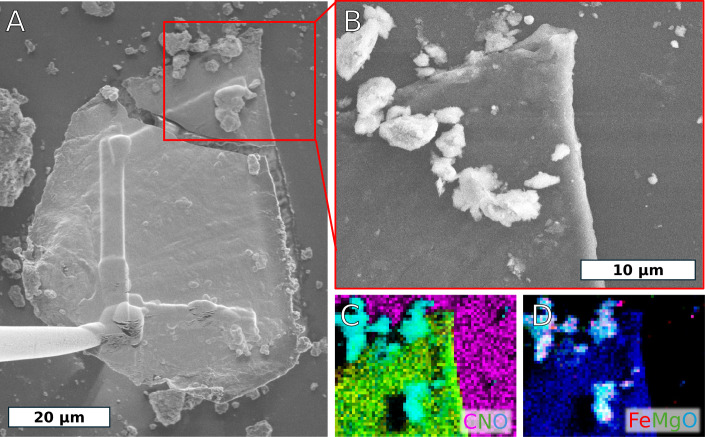
Electron microscopy of Particle 33. **A**, Image of Particle 33 immediately after lifting from diamond substrate. The upper right corner that was measured using SEM/EDS shattered. **B**, SEM image of the region analyzed by EDS. Some phyllosilicates sitting on the surface mobilized during scanning. **C**, C-K (red), N-K (green), and O-K (blue) map showing nitrogen is abundant in Particle 33 but not in the phyllosilicates or diamond substrate. **D**, Fe-L (red), Mg-K (green), O-K (blue) map showing the location of the phyllosilicates (light blue/white), and that Particle 33 contains O but the diamond substrate does not.

**Extended Data Fig. 5:**
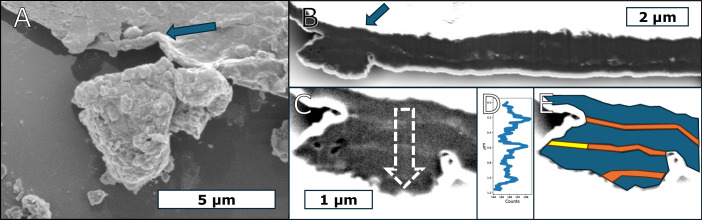
Indications of malleability and layered structures within Particle 33. **A**, A kink in Particle 33 created by pressure against a neighboring phyllosilicate which detached during FIB lift-out. **B**, A FIB cross section of Particle 33 showing the three-layer structure seen in Fig. 3, but on the far-left side is a nodule marked with an arrow containing additional layers. **C**, A zoom in and contrast enhancement of the multilayer nodule. The arrow shows the path and width of an image integration shown in **D**. **D**, An image integration across the arrow in **C** that shows the presence of multiple layers. Bright regions show as peaks going to the right. **E**, A model showing four carbonaceous layers (blue) and three carbonate layers (orange). A yellow region shows a possible extension of the middle carbonate layer through vesicles. It could be that the bottom layers were simply separated and folded over, or that they are truly separate layers.

Some particles have a strong absorption band at 3,285 cm^−1^, which is assigned to N–H stretching (Fig. 1), including Particle 2 from the mixed sample (Figs. 1 and 2) and Particles 26 and 33 from the angular sample (Figs. 1 and 3, Extended Data Figs. 3–5, Supplementary Videos 1 and 2, and Table 1). Particles 31 (N-poor) and 26 (N-rich) are from a single 1-cm stone. We have yet to detect such N-rich organics in the hummocky sample we have studied.

N-rich organics also exhibit aromatic and aliphatic C–H stretching, C=O stretching in amides (R(C=O)NH_*x*_R), N–H bending, CH_2_ bending and C‒O stretching in carbonates, C–N stretching, C–O stretching in aromatic ethers and CH_3_ bending (Table 2 and ref. ^13^, and references therein). A weak band near 2,145 cm^−1^ in the spectra of Particles 2 and 33 may be due to CN stretching in S–C≡N, O–C≡N or N=C=N. A band near 1,740 cm^−1^ for Particle 26 may be esters or lactones. A band around 1,575 cm^−1^ for Particles 26 and 33 could be aromatic C=C modes. A 1,515 cm^−1^ shoulder on the 1,540 cm^−1^ N–H peak is assigned to molecular carbonates.

**Table 2:** IR and STXM results with band and peak identifications

Infrared (Fig. 1)				STXM (Fig. 4)					
Label	Frequency (cm^−1^)	Functional group^a^	STXM^b^		Energy (eV)	Functional group^c^	Upon irradiation or heating	Note	IR^b^
a	3,685	Structural OH in phyllosilicate	N/A	C-K	284.85 (Particle 2)	Aromatic			c
b	3,285	N–H stretch	N-K		285.30 (Particle 33)		Growth, eV increase		
c	3,065	Aromatic C–H stretch	C-K		286.00	C=N and aldehyde (HC=O)		No peak but broad intensity in particle 2	
d	3,000–2,820	Aliphatic C–H stretch	C-K		286.55 (Particle 2)	CH_*x*_ or pyridine		C≡N weak in IR	e
e	2,145 (weak)	CN stretch in S–C≡N, O–C≡N or N=C=N			286.85 (Particle 33)		Growth		
f	1,650	Amide C=O stretch	C-K, N-K, O-K		288.15	CH_*x*_ and amide (R(C=O)NH_*x*_R)	Reduction		b, d, g
g	1,540	N–H bend	N-K		289.45	Urea, alcohol or ether	No effect		j
h	1,465	CH_2_ bend and C–O stretch in carbonates		N-K	398.80	C=N, imine or pyridine	No effect		f
i	1,410	C–N stretch			399.75	Aminated heterocycle	No effect	C≡N excluded from IR	e
j	1,255–1,240	C–O stretch in aromatic ethers and CH_3_ bending	C-K		401.00	Amide	Reduction		b, f, g
k	1,015	Si–O stretch in phyllosilicates	N/A	O-K	531.45	C=O	Unknown		f
					534.50	Alcohol	Unknown		

^a^Functional group assignments for IR are from ref. ^13^ and references therein. ^b^Cross-comparisons between IR and STXM allow for more certain peak assignments. ^c^Functional group assignments for STXM are from refs. ^17,18^, and references therein. N/A, not applicable.

We used the asymmetric and symmetric stretching modes of the –CH_3_ and –CH_2_– groups (Extended Data Fig. 2) to determine the ratio of peak heights of the CH_2_ asymmetric (2,920 cm^−1^) and the CH_3_ asymmetric (2,957 cm^−1^) bands and obtained –CH_2_–/–CH_3_ peak height ratios of 2.4, 2.6 and 3.2 for Particles 33, 2 and 26, respectively. Ratios of 1.0–1.8 are typically observed in CI and CM meteorites and the diffuse interstellar medium, whereas ratios of 2.3–3.0 have been measured for interplanetary dust particles, CR2 meteorites and comet Wild 2 (ref. ^14^). The two symmetric modes of the organic are clearly distinguishable from each other rather than being blended, as is more typical for meteorites.

A greater abundance of –CH_2_– groups indicates longer, less-branched carbon chains. A better estimate of the –CH_2_–/–CH_3_ group ratio were obtained by measuring the areas of these bands and converting into column densities using the average –CH_2_– and –CH_3_ band strengths^15^ of 7.4 × 10^−18^ cm per group and 1.2 × 10^−17^ cm per group, respectively. This resulted in –CH_2_–/–CH_3_ group ratios of 3.1, 4.4 and 5.5 for Particles 33, 2 and 26, respectively (Extended Data Table 1). The presence of terminal functional groups such as NH_2_, COOH, OH or C≡N would also decrease the number of terminal CH_3_ groups in carbon chains and result in higher –CH_2_–/–CH_3_ ratios.

Nano-FTIR spectra of Particle 33, which excludes the presence of adhering phyllosilicates, lack the 1,015 cm^−1^ Si–O stretch, but they exhibit organic peaks consistent with the µ-FTIR spectra and some spectra also show carbonate features (Extended Data Fig. 3).

### Electron microscopy

A focused ion beam (FIB) was used to extract Particle 2 from the diamond window substrate used during IR spectral collection. The grain was placed on an Si substrate (OREX-800107-179), and we prepared an electron-transparent FIB lamella (OREX-800107-180) for TEM and STXM (Extended Data Fig. 1). Similarly, Particle 33 was removed to an Si substrate (OREX-800055-124) and a FIB lamella was produced (OREX-800055-125; Extended Data Fig. 1).

Particle 2 contains a carbonaceous vein sandwiched between phyllosilicates (Fig. 2a,b), which does not otherwise penetrate the phyllosilicate pores. A calcite crystal (CaCO_3_) is in the middle of the vein and vesicles are present, as determined by electron microscopy. The carbonate grain seems to be at the head of a ‘wake’, indicating that it was entrained in a viscous organic flow (Fig. 2a) and existed simultaneously with the viscous organic. A scanning transmission electron microscopy (STEM)/energy-dispersive spectroscopy (EDS) analysis of the carbon vein in Particle 2 gave the composition C:N:O = 3:1:1, indicating extraordinary N and O enrichment (Fig. 2c) compared with insoluble organics in CI and CM meteorites, which typically contain atomic N/C ratios in the 2.5–3.7% range and O/C ratios in the 10–22% range^16^. S, Cl and Ca were detected as minor components (<1 at%). Selected area electron diffraction (Fig. 2d) shows that the carbon is amorphous.

By contrast, Particle 33 is a separate sheet of carbonaceous material on the diamond window after crushing. Some phyllosilicates are associated with it but were not directly adhering as they were in Particle 2 (Extended Data Fig. 4). Optical images of Particle 33 exhibit interference fringes, indicating that it is transparent at optical wavelengths (Extended Data Fig. 2). SEM images show features characteristic of plastic deformation during the flattening process used to prepare the sample for µ-FTIR (Extended Data Fig. 5a). SEM-EDS analysis was performed on one corner of Particle 33 to avoid heavily irradiating the entire object.

A 10 µm × 10 µm × 2 µm region of Particle 33 was sectioned using slice-and-view in the FIB analysis to observe its three-dimensional (3D) structure. The reconstructed data (Fig. 3 and Supplementary Video 2) show that the film is ~2 µm thick and has at least three CNO layers sandwiching two carbonate inclusion layers. The top CNO layer is 2–5 times thicker than the bottom CNO layer. The inclusion layers contain various kinds of crystals, as evidenced by the variable backscatter intensities (α and β in Fig. 3a(i) and Fig. 3f). At least one grain is calcite, and Ca enrichment is present throughout the inclusion layer. In addition, a portion of Particle 33 that was near the slice-and-view volume but not within the 3D reconstruction seems to have more than three layers, possibly up to seven (Extended Data Fig. 5b–e). A low-density inclusion (γ in Fig. 3b(i),f) may be the same material as the surrounding carbon, although it is separated by carbonate. Some of the inclusions, such as δ (Fig. 3c(i),f), have euhedral forms. The composition of Particle 33 as determined by STEM/EDS is C:N:O = 5:1:1 with S, Cl and Ca present at <1 at%. Electron diffraction shows that Particle 33 is amorphous.

### X-ray spectroscopy

**Fig. 4:**
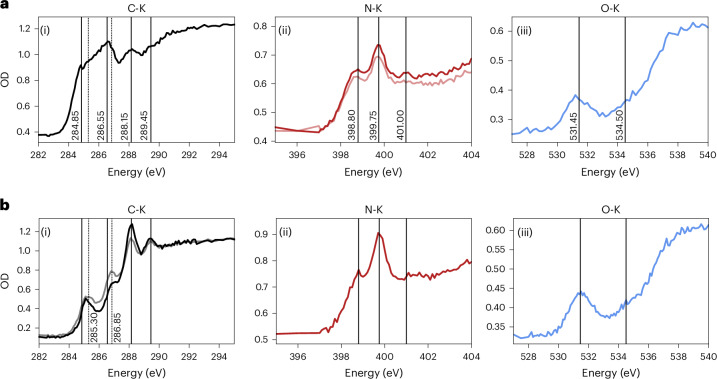
X-ray spectroscopy of the carbon, nitrogen and oxygen K edges of Particles 2 and 33. **a**(i), C-K STXM spectrum of the Particle 2 FIB section showing features at 284.85 eV, 286.55 eV, 288.15 eV and 289.45 eV acquired from a line scan. **a**(ii), N-K STXM spectra of Particle 2 showing peaks at 398.80 eV, 399.75 eV and 401.00 eV. A lighter spectrum shows the result of a second line scan with slightly reduced amplitude and a loss of the 401.00 peak. **a**(iii), O-K STXM spectrum of Particle 2 showing features at 531.45 eV and 534.50 eV. **b**(i), C-K STXM spectra of Particle 33. Features from **a**(i) are marked as well as further positions at 285.30 eV and 286.85 eV. The grey spectrum corresponds to the same region acquired a second time showing loss of the 288.15-eV peak and generation of peaks at 286.85 eV and 285.30 eV. **b**(ii), N-K STXM spectrum of Particle 33. **b**(iii), O-K STXM spectrum of Particle 33. Peak positions are summarized in Table 2.

An STXM analysis of Particle 2 (Fig. 4) shows functional groups in the C-K, N-K and O-K edges that complement the IR analysis (Table 2, refs. ^17,18^ and references therein). A peak at 284.85 eV in the C-K edge indicates C=C bonding. A peak at 286.55 eV could be assigned to C≡N, CH_*x*_ or pyridine, but the low intensity of the 2,145 cm^−1^ band in the IR spectrum discounts abundant C≡N. A peak at 288.15 eV indicates CH_*x*_ or amides (R(C = O)NH_*x*_R). A weak peak at 289.45 eV may indicate urea, alcohol or ether groups. A poorly resolved peak between 285 eV and 286 eV could be due to the presence of C=N or C=O. In the N-K edge, peaks are present at 398.80 eV, 399.75 eV and 401.00 eV, which we assign to C=N and pyridine, aminated heterocycles, and amide, respectively. A second line scan over the same location showed a reduced overall intensity of N, and the amide peak disappeared. We ascribe this to the beam sensitivity of the N–H bonds in amines and amides. The O-K spectrum shows peaks at 531.45 eV and, faintly, 534.50 eV, which we assign to C=O and ether or alcohol bridges, respectively.

STXM spectra of Particle 33 (Fig. 4) exhibit different features in the C-K edge compared with Particle 2. The lowest-energy peak at 285.0 eV (C=C) had shifted 0.1–0.2 eV higher, perhaps due to the loss of aromatic H or alkyl side chains. A second line scan of C-K at the same location caused the C=C peak to shift further to 285.30 eV with increased intensity. A peak at 286.85 eV was 0.3 eV higher than the comparable peak in Particle 2 and increased in intensity in the second C-K line scan. We assign this peak to CH_*x*_ and pyridine. Both were probably present before our STXM analysis, and extra pyridine was generated during the analysis from loss of H near C=N bonds. A peak at 288.12 eV (CH_*x*_ or amide) dropped in intensity on the second line scan. A fourth peak at 289.17 eV (alcohol, urea and ether groups) was more pronounced than in Particle 2 and did not change between line scans. The changes in C-K spectra demonstrate the loss of H from CH_*x*_ and amide and the creation of pyridine and C=C due to irradiation. The N-K and O-K spectra have the same peak assignments as Particle 2.

### Isotopes

Nitrogen isotopic measurements of Particle 33 using SIMS yielded δ^15^N_air_ = −28 ± 7‰ (2σ) anchored to a 3.5-billion-year-old terrestrial kerogen standard from Australian Warrawoona group chert (sample PPRG 002)^19^.

### Water solubility

**Extended Data Fig. 6:**
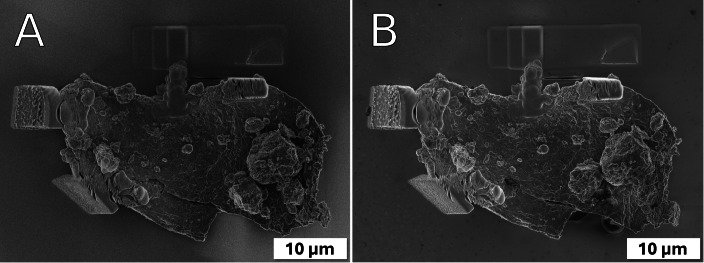
SEM images of Particle 64 welded to an Au-coated Si chip with Pt in a FIB. **A**, Before immersion in deionized water. **B**, After immersion in deionized water. No significant changes are apparent. Some phyllosilicates adhered to the surface of the sheet have moved in the process.

To determine whether the N-rich organic is water-soluble, we moved another N-rich organic grain from the angular lithology OREX-800055-123 (Particle 64) from the diamond window to an Au-coated Si chip (Extended Data Fig. 1) and immersed it under a droplet of deionized water from a hypodermic syringe. After the water had evaporated, the grain was unchanged (Extended Data Fig. 6).

## Discussion

The N- and O-enriched carbonaceous materials contain both aromatic and aliphatic C‒H bonds, with aliphatics dominated by CH_2_ over CH_3_ groups. This favours longer chains or cycles over branched aliphatics. The N is present as N–H (amines and amides), C=N and N-heterocycles. The O is present as C=O and as O–H-containing moieties (Table 2).

### Organic formation

Based on the N-rich chemistry reported in soluble organics, components of Bennu’s parent body accreted beyond the ammonia ice line^12^. Both observations and models indicate that the composition of ices in protostellar nebulae can vary dramatically. Both H_2_O-dominated ices containing CO_2_ or NH_3_ in small concentrations or distinct CO_2_- and NH_3_-dominated ices are possible depending on the conditions^20,21^. H_2_O-dominated ice can form a eutectic with NH_3_ as low as 176 K, depending on pressure and impurity concentration^22^. NH_3_- and CO_2_-dominated ices sublime well below this eutectic point and can react chemically, whereas H_2_O-dominated ice remains frozen^23,24^.

In the earliest stages of the parent body’s existence, radioisotope decay produced heat, which caused an increase in temperature. Under these conditions, NH_3_- and CO_2_-dominated ices melt or sublime before H_2_O, irrespective of any NH_3_ concentration in the H_2_O-dominated ice. A mixture of NH_3_ and CO_2_ with excess NH_3_ produces ammonium carbamate (NH_4_COONH_2_) at 75 K (ref. ^25^). As the temperature increases above ~150 K, the carbamate converts to carbamic acid until H_2_O melts, at which point the carbamic acid decomposes into ammonium (NH_4_^+^) and carbonate (CO_3_^2−^). Carbamate can be a polymerization precursor for amine and amide polymers^26^ and may be a monomer of the N-rich organic polymeric material we observed. Indeed, experimental evidence under relevant conditions indicates that the presence of small amounts of H_2_O ice can catalyse carbamate formation^27^. Once H_2_O ice melts and any unpolymerized carbamate decomposes, NH_4_^+^ would diffuse throughout the parent body, and it would be difficult or impossible to reach the high concentration of N relative to C necessary to produce N-rich organic materials such as we see. Therefore, the initial polymerization of carbamate to produce Particles 2 and 33 probably occurred at temperatures below the melting point of H_2_O ice, and any unpolymerized monomer decomposed into NH_4_^+^ and CO_3_^2−^ after the H_2_O ice had melted.

Late-stage evaporation of water brines on Bennu produced an evaporite sequence of carbonates, sulfates, phosphates and other salts, from which the evolution of pH can be inferred^28^. Calcite is the earliest phase formed in the evaporation sequence at <10% brine evaporation. At this point, the pH of the fluid was close to neutral. The incorporation of calcites within and on the carbonaceous sheets described here implies further evolution of the organic material after the production of the calcites.

**Extended Data Fig. 7:**
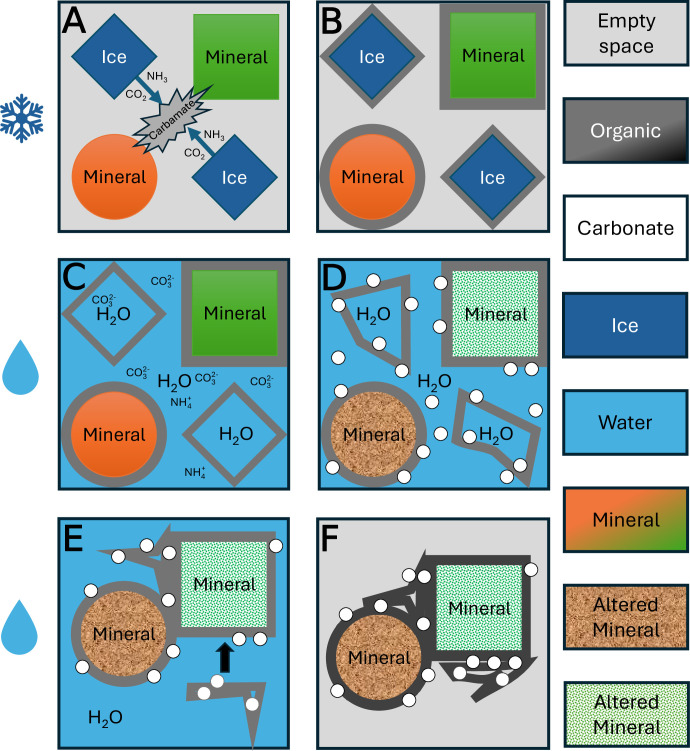
Proposed multistep formation process of the organic phase. **A**, As the parent body warmed, NH_3_ and CO_2_ from ices reacted to form carbamate via gas–grain or solid-state reactions before H_2_O ice melted. **B**, Carbamate underwent partial polymerization to produce a water-insoluble organic phase at low temperatures that formed or was deposited on grain surfaces (gray). **C**, Further warming melted H_2_O ice and decomposed any unpolymerized carbamate into NH_4_^+^ and CO_3_^2−^ dissolved in water (blue). **D**, Aqueous alteration led to the precipitation of some calcites (white circles) and initiated conversion of anhydrous silicates to phyllosilicates (textured fills of the circle and square). Calcites precipitated as either isolated grains or grains that stuck to or nucleated on the organic surfaces. **E**, Organic coatings from adjacent grains adhered, producing a layered structure with embedded carbonates and voids. Any mantles around ice grains would collapse or fold. **F**, Further aqueous alteration may have then folded the polymer sheets to produce more complicated structures, triggered additional chemistry, and set the final morphology (dark gray). Panels **A** and **B** occur at cryogenic temperatures below the melting of the H_2_O–NH_3_ eutectic, whereas panels **C–F** occur after the melting of H_2_O ice.

Therefore, we propose a multistep chemical process (Extended Data Fig. 7): (1) As the parent body warmed, NH_3_ and CO_2_ from ices reacted to form carbamate through gas–grain or solid-state reactions before H_2_O ice melted (Extended Data Fig. 7a). (2) Carbamate underwent partial polymerization to produce a water-insoluble phase at low temperatures that formed or was deposited on grain surfaces (Extended Data Fig. 7b). (3) Further warming melted H_2_O ice and decomposed any unpolymerized carbamate into NH_4_^+^ and CO_3_^2−^ (Extended Data Fig. 7c). (4) Aqueous alteration of minerals created some calcites and initiated phyllosilicate conversion. Some calcites stuck to or nucleated on the organic surfaces (Extended Data Fig. 7d). (5) Organic coatings from adjacent grains adhered, producing a layered structure with embedded carbonates and voids. Any mantles around ice grains would collapse or fold (Extended Data Fig. 7e). (6) Further aqueous alteration may have then folded the polymer sheets to produce more complicated structures, triggered further chemistry and set the final morphology (Extended Data Fig. 7f).

Although CO_2_ and NH_3_ are common in primitive icy bodies^29^, it is unclear how NH_3_ was distributed across Bennu’s entire parent body. The presence of nitrogen-rich organics in only certain lithologies indicates either that localized chemical conditions were necessary for their formation or that they were destroyed during subsequent alteration stages. Because we found this phase only in the angular sample and the mixed sample (which presumably contained angular particles) but have yet to find it in the hummocky sample, we conclude that the angular lithology formed in a portion of Bennu’s parent body with excess NH_3_ relative to CO_2_ and conditions suitable for polymerization reactions. By contrast, either conditions in the hummocky lithology may not have favoured these polymerization reactions or the hummocky lithology experienced processes that subsequently destroyed this phase.

### Nitrogen isotopes

The δ^15^N isotopic value near −30‰ is within the range of N isotopic values seen in Bennu spot analyses^30^. In addition, two ‘cold spots’ with δ^15^N values near −30‰ have been seen in nano-SIMS analyses of organics in samples from the carbonaceous asteroid Ryugu^31^, and six cold spots between −50‰ and −200‰ have been seen in the CI chondrite Orgueil^32^. It is possible that the nitrogen cold spots measured in Ryugu and Orgueil are analogous to the N-rich organic material seen here.

Negative δ^15^N values are not as commonly seen in extraterrestrial organics as positive δ^15^N anomalies. Nitrogen in soluble Bennu extracts show positive δ^15^N values^12^. As the N-rich organic materials we found contain negative δ^15^N, different chemical processes may have been involved in their formation compared with the soluble organics reported by Glavin, Dworkin and colleagues^12^. Various processes have been suggested for producing positive δ^15^N values in extraterrestrial materials, including ion–molecule reactions and aqueous parent-body chemistry involving heavy δ^15^N precursors^16^. Ion–molecule reactions at very low temperatures favour heavy nitrogen^33^. Equilibrium fractionation reactions at moderate to high temperatures can produce only minor mass-dependent fractionation and, therefore, require ^15^N-rich precursors to produce high δ^15^N values. By contrast, the observed negative δ^15^N in the materials studied here is more consistent with kinetic fractionation chemistry at cryogenic temperatures, which favours light isotopes in the chemical products^34^.

### Emplacement evidence

The particles we studied place constraints on the physical emplacement processes of the organic sheets that complement the chemical sequence described above.

The 3D reconstruction of Particle 33 (Fig. 3) indicates that the layers could have formed through deposition associated with chemical steps (4)–(6). The physical and chemical heterogeneity of particle types (α, β and δ in Fig. 3) in the middle layer of Particle 33 is consistent with the idea of particulates decorating organic coatings on pre-existing grains. The subsequent movement of coated grains caused some of them to adhere to each other and sandwich the carbonates between organic sheets. Such reworking could also detach sheets and allow them to fold or stack to produce multilayered structures, as seen in Particle 33 (Extended Data Fig. 5c). The convoluted shape of the organic vein in Particle 2 with a carbonate and void inclusions could be explained by such folding (Fig. 2a). In addition, small portions of detached organic material could deposit onto other organic coatings and explain features such as γ, the organic inclusion in the carbonate mid-layer (Fig. 3).

### Implications for prebiotic chemistry

The observation of the N- and O-rich phases reported here, combined with the detection of N-rich soluble organic compounds^12^, demonstrates that asteroids like Bennu’s parent body could have been a substantial source of N-rich volatiles and compounds of biological importance, including ammonia, amino acids, nucleobases and other chemical precursors that contributed to the prebiotic inventory that led to the emergence of life on Earth. The observation of polymeric N-rich organics that contain abundant amine and amide functional groups adds a new dimension to this asteroidal prebiotic inventory. Carbamates can be readily formed from NH_3_ and CO_2_ at temperatures relevant to astrophysical conditions and are known precursors to prebiotic compounds including urea and amino acids^27,35–39^. Carbamates could have played a role in the formation of amino acids on early Earth^40^. The polymerization of carbamates results in the formation of materials with molecular bonds like those found in peptides (amide groups), nucleobases (N-heterocycles) and polyols (alcohols and carbonyls), as supported by IR and STXM spectra (Figs. 1 and 4 and Table 2), that are potential precursors to biologically relevant compounds once they are seeded on the surface of a planet.

## Methods

The samples collected from asteroid Bennu used in this investigation were stored in an inert atmosphere before being analysed. Therefore, they retained reactive organic phases that could be present in the original body but possibly would not be found in meteorites that have experienced atmospheric entry and weathering. Indeed, some carbonaceous phases are sensitive to oxidation or weathering in a terrestrial environment, so a pristine sample is crucial for reliable organic analyses of reactive phases. Such studies have been practical previously only with Ryugu samples^41,42^.

### Samples used in this investigation

The individual particles studied in this work are listed in Extended Data Fig. 1. They include particles taken from powdered Bennu samples OREX-800107-105 (97.4 mg) and OREX-800055-107 (1.4 mg), which were splits taken from a powdered aggregate sample (where ‘aggregate’ refers to a mixture of particles <0.5 cm, unsorted by lithology; OREX-800107-0, 6.425 g), plus a chip from the angular lithology (OREX-800055-3, 87.2 mg), that were crushed in air using a quartz mortar and pestle inside a HEPA-filtered laminar flow bench. Individual particles from powdered Bennu sample OREX-800088-102 (0.9 mg), which was a split taken from a hummocky sample (OREX-800088-3, 39.4 mg), were also studied, but are not discussed further here as no organics of the type described in this work were found in this sample. The materials and processes used to make these powders are described elsewhere^12^.

Small (~0.1 mg) splits of OREX-800107-105 and OREX-800055-107, namely, OREX-800107-178 and OREX-800055-123, respectively, were mounted on the diamond windows used for our µ-FTIR measurements. The individual Particle 2 (from OREX-800107-178) and Particles 33 and 64 (from OREX-800055-123) were subsequently removed from their diamond substrates for further analyses, including EDS, STXM, TEM, nano-FTIR and water immersion.

### µ-FTIR spectroscopy

At NASA Ames Research Center, samples were prepared for µ-FTIR analysis by taking ~0.1 mg of powdered Bennu samples OREX-800107-105 (aggregate of mixed lithologies), OREX-800055-107 (angular lithology) and OREX-800088-102 (hummocky lithology) and placing them between clean sapphire disks (25 mm in diameter and 1 mm thick). These plates were then pressed together to crush and disperse the samples. This process created particles ranging in size from submicrometres up to ~50 µm in diameter. Portions of this crushed material were then transferred to clean diamond squares (5 mm × 5 mm and 0.5 mm thick) by simple contact. Diamond windows with adhering Bennu material were then mounted on a special holder that could be placed on the stage of a Thermo Fisher Nicolet iN10 MX FTIR microscope. This process generated a field of Bennu particles suitable for µ-FTIR analysis. The microscope was equipped with a conventional Globar light source and a liquid N_2_-cooled mercury-cadmium-telluride detector capable of measuring transmission spectra in the mid-IR range (4,000–675 cm^−1^, 2.5–14.8 µm) at a spectral resolution of 4 cm^−1^. Spectra of individual particles in the 10–50 µm-diameter range were collected by averaging several interferometer scans, typically between 512 and 4,096 scans.

### Nano-FTIR spectroscopy

Nano-FTIR measurements were performed on the synchrotron infrared nanospectroscopy (SINS) beamline 5.4 at the Advanced Light Source in Berkeley, California^43,44^. The SINS spectra of Particle 33 were collected at 8 cm^−1^ spectral resolution using a gold-coated atomic force microscope tip (NanoAndMore, PPP-NCHAu-MB-10) oscillating at ~250 kHz with a tapping amplitude of ~80 nm in a modified atomic force microscope (Bruker Innova) coupled to an FTIR spectrometer (Thermo Fisher Scientific Nicolet 6700) with a potassium bromide (KBr) beam splitter and a mercury-cadmium-telluride detector (Kolmar). The data shown are the Fourier-transformed *n* = 2 phase, *ϕ*_2_(*ω*), spectra of the complex optical *S*_*n*_(*ω*) = *s*_*n*_(*ω*) e^i*ϕn*(*ω*)^ signal. The spectra are all referenced to the silicon substrate and a gold reference.

### Focused ion beam

Particles 2 and 33 were removed from their respective diamond IR substrate windows using an FEI Helios G4 UX dual-beam FIB-SEM at the Molecular Foundry within the Lawrence Berkeley National Laboratory in Berkeley, California. The samples were first coated with a thin (5–10 nm) carbon film using a sputter coater. They were imaged using electron beam energies between 1 keV and 10 keV and currents of ~100 pA. EDS mapping was carried out at ~500 pA. The samples were then welded to a tungsten needle and subsequently transferred and welded through flying buttresses to an Si substrate with deposited Pt (trimethyl(methylcyclopentadienyl)platinum(IV)). To ensure the grains remained intact during transfer, Pt straps and a Pt layer were painted across the particles before lifting. All ion-beam work was done with a Ga^+^ ion beam at energies of 16 keV or below. Rough cutting was done at 16 keV and 8 keV, and cleaning was done at energies down to 1 keV. In addition, currents were kept at ~5 nA or less at all steps to minimize heating damage, and electron imaging during FIB preparation was kept at energies ≤1 keV to reduce heating damage^45^.

Particle 2 was mounted on a copper grid by cutting a notch at the top of a Cu grid post and welding it on each side and then thinned to ~200 nm to preserve the vein structure. It was transferred to the TEM stage for the low-dose analysis, which was followed by STXM and subsequent return to the TEM stage for a high-dose analysis.

Particle 33 was mounted on a copper grid in the flag style as a 10 µm × 2 µm × 10 µm object. It was then thinned in 100-nm increments to produce a 10 µm × 2 µm × 0.1 µm object. Backscatter images were recorded at each slice and reconstructed to produce a 3D view. It was then transferred for STXM and TEM.

A patch was milled on the side of Particle 33 using Ga^+^ ion milling to provide a region free of Pt deposition for nano-FTIR. The similarity in the functional groups imaged in nano-FTIR and far-field IR (before any FIB work) indicates that the FIB process was gentle enough to preserve the carbonaceous composition and molecular structure and that no residual Pt deposition contaminated the nano-FTIR spectrum.

### Transmission electron microscopy

The TEM analysis was done on an FEI TitanX microscope at beam energies of 80–300 keV. STEM/EDS maps were acquired at 80 keV as a sequence of maps starting at low beam currents of ~10 pA and increasing to 100 pA using a 0.6 sr Bruker quad silicon drift detector. To control the volatilization of light elements, especially hydrogen and loosely bound oxygen, maps were acquired sequentially for periods of several minutes to ~0.5 h and then combined using Python. Elemental quantification for CNO was standardized using HN-Kapton purchased from McMaster Carr. The Kapton was analysed by FIB using the same protocols as used for Particles 2 and 33 but with a wedge shape to accommodate the varying thickness of the material. The Kapton was DuPont HN polyimide film, which has a chemical formula of (C_22_H_10_N_2_O_5_)_*n*_. The bremsstrahlung for the Kapton was modelled using DTSA II^46^, and the peaks of the experimental function were fitted with Gaussians, sigmoids and a line to match the general shape of the modelled bremsstrahlung and experimental data. The same model was used to fit the equivalent peaks for Particles 2 and 33. Quantification was done using Stoichiometry Fitter^47^. Imaging and diffraction were done at 80 keV or 300 keV and at beam currents lower than 0.25 nA.

### Scanning transmission X-ray microscopy

Synchrotron studies were carried out at the STXM beamline 5.3.2.2 of the Advanced Light Source at Lawrence Berkeley National Laboratory to measure X-ray absorption spectra^48^. Beamline 5.3.2.2 uses a spherical grating monochromator for which calibration involves two parameters: the zero of the grating motor and the included angle between the entrance and exit arms. The former was calibrated using the specular (zeroth-order) beam and the latter using the sharp absorption peak exhibited by CO_2_ gas at 292.74 eV (ref. ^49^). Line scans were acquired from FIB sections using an X-ray beam focused using a Fresnel zone plate down to several tens of nanometres and by rastering the sample at various beam energies to produce data with one spatial dimension and one energy dimension. The edges studied included the C-K, N-K and O-K edges. The spatial steps were typically of the order of the spot resolution, and the energy steps ranged from 0.1 eV to several electronvolts depending on the expected presence of features in the spectrum. The dwell times were between 3 ms and 15 ms, where the 5-ms dwell times were used for initial acquisition, and the 5-ms to 15-ms dwell times were used later to improve the signal-to-noise ratio and to monitor for potential beam damage. In one case, a 3-ms dwell time was used when it became apparent that the N-K edge was beam sensitive. Because of the beam-sensitive character of the samples, defocused line scans were collected on neighbouring regions on the assumption that the sample was homogeneous or nearly homogeneous. Two line scans were collected sequentially on the same line in Particle 2 for the C-K edge to quantify the beam sensitivity of carbonaceous functional groups. Similarly, two line scans were collected sequentially on the same line in Particle 33 for the N-K edge to quantify the beam sensitivity of N-bearing functional groups. The data were reduced to the optical density and analysed to identify functional groups^50^.

### Secondary-ion mass spectroscopy

SIMS measurements were done using the Cameca 7f-GEO ion probe at Washington University in St Louis. A 20-pA primary beam focused to 3 µm was used to pre-sputter FIB-deposited organometallic Pt from the surface of Particle 33 by rastering a 5 µm × 5 µm area while monitoring C, CN and O. After approximately 45 min, a sudden increase in CN/C indicated that the pre-sputtering action was complete. The primary beam current was decreased to 3 pA and focused to ~1.5 µm with a 5 µm × 5 µm raster. ^12^C^14^N and ^12^C^15^N were collected with a mass-resolving power of 5,000. The CN counts decreased after 2 h and coincided with a sudden homogeneous Si signal, indicating that the sputtering process had penetrated the sample. Kerogen from chert (Warrawoona group, Australia, 3.5 billion years old) was used as an N isotope standard. This kerogen contains 64 wt% of C and has a C/N wt% ratio of 181.59, H/C ratio of 0.3, δ^13^C_PDB_ = − 34.3‰ and δ^15^N_air_ = 5.5‰ (PPRG 002)^19^.

## Supplementary information

Supplementary Video 1 Slice-and-view slice sequence of Particle 33. Imaging at 2 keV, 25 pA, secondary electron imaging, 52° tilt and 4 mm working distance.

Supplementary Video 2 Rotating movie of the reconstruction of the slice and view of Particle 33.

## Figures and Tables

**Extended Data Table 1:**
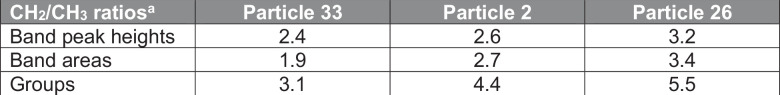
The relative abundance of CH_2_ and CH_3_ groups in Particles 2, 26, and 33

^a^The CH_2_/CH_3_ ratios in these three particles can be examined using three different approaches after fitting the four bands in the 3000–2820 cm^−1^ region: (1) ratios from the peak heights of the CH_2_ and CH_3_ asymmetric stretching bands, (2) similar ratios from the areas of the same peaks, and (3) after converting the areas of the CH_2_ and CH_3_ asymmetric stretching bands into column densities of –CH_2_– and –CH_3_ groups, using the average values of the band strengths from ref. ^15^ of 7.4 × 10^−18^ and 1.2 × 10^−17^ cm group^−1^ for –CH_2_– and –CH_3_ groups, respectively.

**Extended Data Table 2:**
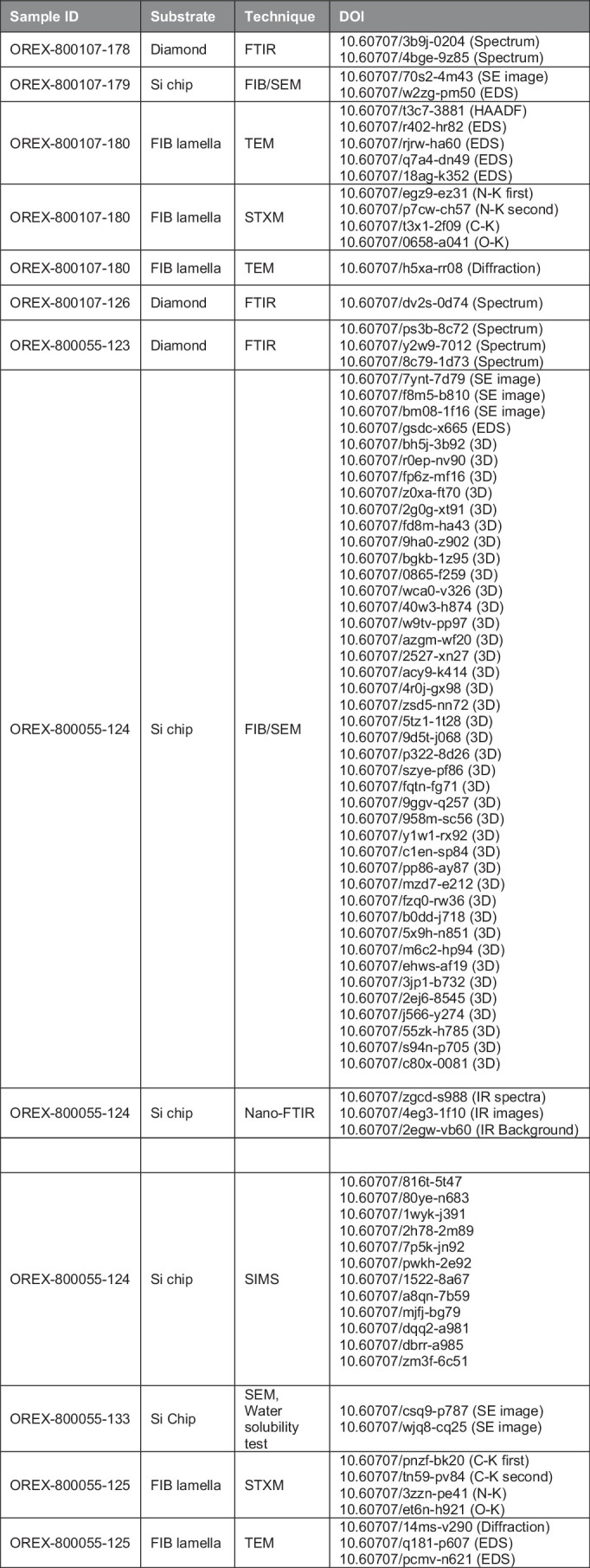
DOIs of sample analysis data products

a) FTIR, Fourier Transform Infrared Spectroscopy; FIB, Focused Ion Beam; SEM, Scanning Electron Microscopy; TEM, Transmission Electron Microscopy; Nano-FTIR, Nanoscale Fourier Transform Infrared Spectroscopy; SIMS, Secondary Ion Mass Spectroscopy; STXM, Scanning Transmission X-ray Microscopy.

## Data Availability

The instrument data supporting the experimental results in this study are available at http://astromat.org/ at the DOIs given in Extended Data Table 2.
