# Supplementary figures and images for: Nitrogen- and oxygen-rich organic material indicative of polymerization in pre-aqueous cryochemistry on Bennu’s parent body
Source: Nat Astron. 2025 Dec 2;9(12):1803–11. doi: 10.1038/s41550-025-02694-5 (PMC12708357; doi:10.1038/s41550-025-02694-5)

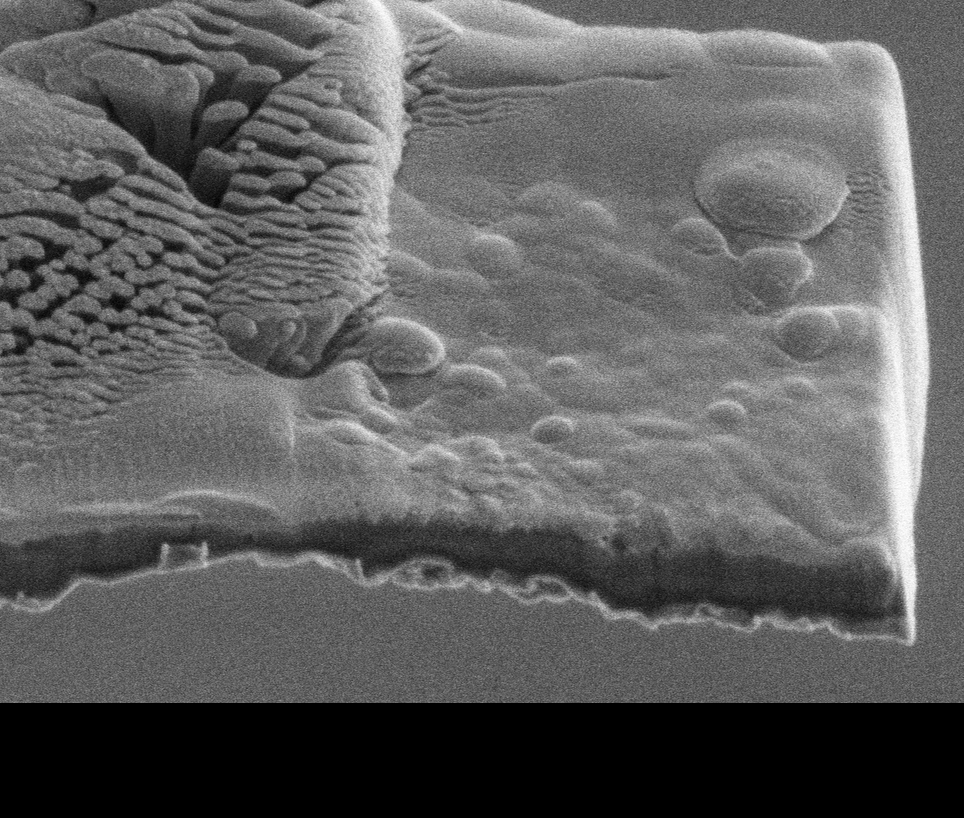

Supplement: Supplementary file 1 — Slice-and-view slice sequence of Particle 33. Imaging at 2 keV, 25 pA, secondary electron imaging, 52° tilt and 4 mm working distance. [file 41550_2025_2694_MOESM1_ESM.gif]
